# Non-Alcoholic Fatty Liver Disease and Bone Tissue Metabolism: Current Findings and Future Perspectives

**DOI:** 10.3390/ijms24098445

**Published:** 2023-05-08

**Authors:** Oxana M. Drapkina, Anastasia Yu. Elkina, Anna F. Sheptulina, Anton R. Kiselev

**Affiliations:** 1 Department of Fundamental and Applied Aspects of Obesity, National Medical Research Center for Therapy and Preventive Medicine, 101990 Moscow, Russia; 2 Department of Therapy and Preventive Medicine, A.I. Evdokimov Moscow State University of Medicine and Dentistry, 127473 Moscow, Russia; 3 Department of Intermediate Level Therapy, Saratov State Medical University, 410012 Saratov, Russia; 4 Coordinating Center for Fundamental Research, National Medical Research Center for Therapy and Preventive Medicine, 101990 Moscow, Russia

**Keywords:** non-alcoholic fatty liver disease, bone tissue metabolism, osteoporosis

## Abstract

Non-alcoholic fatty liver disease (NAFLD) is reaching epidemic proportions worldwide. Moreover, the prevalence of this liver disease is expected to increase rapidly in the near future, aligning with the rise in obesity and the aging of the population. The pathogenesis of NAFLD is considered to be complex and to include the interaction between genetic, metabolic, inflammatory, and environmental factors. It is now well documented that NAFLD is linked to the other conditions common to insulin resistance, such as abnormal lipid levels, metabolic syndrome, and type 2 diabetes mellitus. Additionally, it is considered that the insulin resistance may be one of the main mechanisms determining the disturbances in both bone tissue metabolism and skeletal muscles quality and functions in patients with NAFLD. To date, the association between NAFLD and osteoporosis has been described in several studies, though it worth noting that most of them included postmenopausal women or elderly patients and originated from Asia. However, taking into account the health and economic burdens of NAFLD, and the increasing prevalence of obesity in children and adolescents worldwide, further investigation of the relationship between osteopenia, osteoporosis and sarcopenia in NAFLD, including in young and middle-aged patients, is of great importance. In addition, this will help to justify active screening and surveillance of osteopenia and osteoporosis in patients with NAFLD. In this review, we will discuss various pathophysiological mechanisms and possible biologically active molecules that may interplay between NAFLD and bone tissue metabolism.

## 1. Introduction

Non-alcoholic fatty liver disease (NAFLD) is one of the pathogenetic determinants of metabolic syndrome [1,2]. In recent years, the NAFLD concept has changed substantially. Whereas previously such pathology was considered just a manifestation of metabolic syndrome, there is currently convincing evidence that NAFLD is one of the key factors in its development [3]. In addition, the results of many studies conducted over recent decades have demonstrated that NAFLD has a wide range of extrahepatic manifestations, for e.g., conclusive data were obtained on the close relationships between NAFLD and type 2 diabetes mellitus (T2DM) [4], chronic kidney disease [5], atherosclerosis, and cardiovascular diseases [6]. Some publications confirmed a higher prevalence of cardiomyopathies, cardiac arrhythmias, osteoporosis, obstructive sleep apnea syndrome, polycystic ovary syndrome, psoriasis, hypothyroidism, urolithiasis, and periodontal disease in patients with NAFLD [6,7,8]. In this regard, the adverse prognostic value of NAFLD for the patient seems quite obvious: this liver disease is associated with an increase in morbidity and mortality. In particular, it is well-known that the presence of NAFLD entails a fivefold, or even a tenfold, risk of death from cardiovascular pathology and other causes, and 19% of deaths in NAFLD are due to malignant neoplasms of extrahepatic origin [8].

Considering the contemporary belief that NAFLD is a systemic metabolic pathology, as well as the lack of effective treatment methods for this ailment (only 20–30% of patients experience a reduction in the severity of non-alcoholic steatohepatitis [NASH] and/or regression of liver fibrosis), it is crucial to clarify the mechanisms, risk factors, and markers of NAFLD pathogenesis. Accordingly, the attention of an increasing number of researchers in recent years has been drawn to the problem of the relationship between NAFLD and bone tissue pathology, some aspects of which are discussed in this review.

## 2. Methods

The objective of this literature review was to identify evidence that demonstrates the relationship between the development and progression of NAFLD and the disturbances in bone tissue metabolism, with a focus on the most recent data published within the past 5 years. In this regard, we searched the PubMed electronic database on 6 February 2023 using the following PubMed filters:

- Article type: Classical Article, Clinical Study, Clinical Trial, Comparative Study, Controlled Clinical Trial, Multicenter Study, Meta-Analysis, Observational Study, and Randomized Controlled Trial, Preprint;
- Species: Humans, Other Animals;
- Article language: English;
- Age: Child, Adolescent, Adult;
- Publication date: 5 years.

Articles outside of the PubMed search were also included, if relevant.

Articles were included in the full-text review in cases where they reported on disease burden, epidemiology, pathogenic mechanisms of the relationship between NAFLD and bone density, the association of bone tissue metabolites with the development and progression of NAFLD, as well as the effects of treatment with bone tissue metabolites, their analogs, and agonists/antagonists of their receptors on the metabolic disturbances associated with NAFLD and/or on liver inflammation and fibrosis in NAFLD.

## 3. NAFLD and Decreased Bone Mineral Density: Some Epidemiological Evidence

There are published data on the relationship between the NAFLD pathogenesis and bone remodeling processes. Associations were established between the presence of NAFLD and a reduction in bone mineral density (BMD), myosteatosis, sarcopenia, and an augmented risk of osteoporosis [6,7,8,9]. In turn, such pathological processes (myosteatosis, sarcopenia, and disorders of bone tissue metabolism) may be associated with an aggravated course of NAFLD. Some studies established that bone turnover in elderly patients with NAFLD was associated with a slowdown in the rate of bone formation and increased rate of bone resorption, which was indicative of an amplified risk of developing osteoporosis in this cohort [10,11,12].

A study conducted in Taiwan revealed that patients with NAFLD were more likely to suffer from osteoporosis than patients in the control group. In addition, it was found that the risk of developing osteoporosis in patients with hepatic steatosis was 1.35 times higher [6]. In a study conducted in East China, the combined effect of NAFLD and metabolic syndrome on osteoporosis in postmenopausal women was well documented. For example, the average BMD in the lumbar vertebrae was significantly lower in patients with NAFLD vs. women without this pathology. The researchers attributed these findings to estrogen deficiency. The latter plays a protective role in the development of osteoporosis by indirectly inhibiting osteoclast activity via increasing the activity of osteoblasts and suppressing the effects of proinflammatory cytokines: interleukins (IL-1 and IL-6), tumor necrosis factor alpha (TNF-α), and macrophage colony-stimulating factor (M-CSF) [12].

Despite the factthat osteoporosis is known to be most common among postmenopausal women, few studies have been published investigating the possible association between NAFLD and osteoporosis in men. They are of particular interest since NAFLD is regarded as a sexual dimorphic disease with a higher prevalence in men. One of such studies, performed by Hye Jun Lee et al. (2021), demonstrated an association between the 10-year likelihood of fracture and the presence of NAFLD in Korean men with or without sarcopenia. For instance, the 10-year probability of large osteoporotic fractures was significantly higher in the group of patients with NAFLD aged ≥50 years (*p* = 0.002), and this direct relationship was more pronounced in patients with sarcopenia. Low muscle mass was associated with the histological severity of NAFLD, while sarcopenia was significantly associated with non-alcoholic steatohepatitis (NASH) and advanced fibrosis, independent of obesity, inflammation, and insulin resistance [13]. In another study Zhe Shen et al. (2020) showed that the association between NAFLD and development of low BMD was similarly observed in both men and women, whereas the association between NAFLD and incidence of low BMD tended to be stronger in men compared to women [14]. Thus, the above-mentioned data suggest that other mechanisms besides estrogen deficiency may underlie the relationship between NAFLD and low BMD, and some of them will be discussed in more details in this review.

Pan Binjing et al. (2022) performed a systematic review and meta-analysis to investigate the relationship between the prevalence and risk of osteoporosis or osteoporotic fractures and NAFLD. They discovered that the prevalence of osteoporosis or osteoporotic fractures was higher in the group of patients with NAFLD compared with the group of patients without it [odds ratio (OR) = 1.17, 95% confidence interval (CI): 1.04, 1.31] [15].

We found only a few studies on the relationship between osteosarcopenia and NAFLD in a cohort of young and middle-aged patients, since osteopenia, osteoporosis, and/or sarcopenia were traditionally considered diseases occurring at an older age. One of such studies demonstrated that NAFLD had a negative impact on BMD in obese adolescents, probably due to insulin resistance [16]. Additionally, NAFLD and obesity can also impair bone mineralization in children, drawing attention to potential risk factors for osteoporosis other than ageing. An Italian case–control study of 44 obese children diagnosed with NAFLD revealed a significantly lower BMD Z-score in the lumbar spine in these children vs. children without NAFLD. In a cohort of children with obesity and NAFLD (a liver biopsy was performed to diagnose NASH and the stage of liver fibrosis), it was shown that children with NASH had a significantly lower BMD Z-score of the lumbar vertebrae, compared with children with simple steatosis, which implied the role of mild inflammation in the process of bone loss [17]. Similar results were obtained in another study in 38 children with biopsy-confirmed NAFLD. More pronounced bone demineralization was detected in children with non-alcoholic steatohepatitis vs. those with simple steatosis [18].

Most studies on examining the relationship between NAFLD and bone pathology were carried out in the countries of the Asia-Pacific region and were conducted on elderly patients, while information on this issue in young and middle-aged people was not covered enough in the published sources. Furthermore, virtually no data are available on the prevalence of osteopenia and osteoporosis in a cohort of patients with NAFLD in the European region.

## 4. Pathogenetic Mechanisms of the Relationship between NAFLD and Reduced Bone Mineral Density

**Figure 1:**
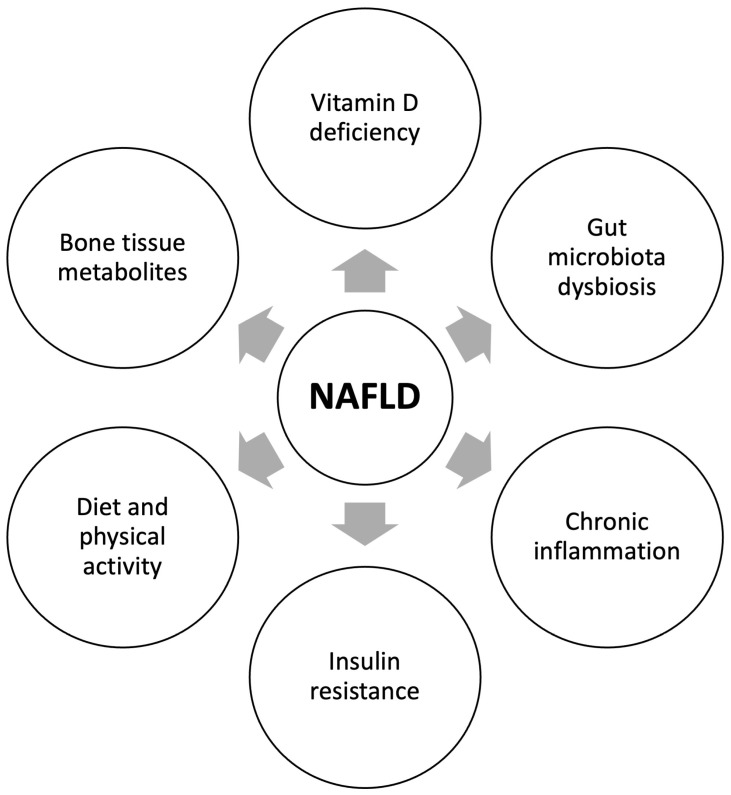
The key pathogenic mechanisms underlining the relationship between NAFLD and reduced bone mineral density. The pathogenesis of decreased BMD in NAFLD appears to be multifactorial and includes the following mechanisms: vitamin D deficiency, chronic inflammation, insulin resistance, and gut microbiota dysbiosis, leading to increased gut barrier permeability, changes in bile acids signaling, as well as the content and profile of short-chain fatty acids. The Western diet and decreased physical activity can also play a role. Finally, bone tissue metabolites due to their impact on the lipid and glucose homeostasis, as well as bone turnover and liver fibrosis, may contribute to decreased BMD in NAFLD.

Figure 1 summarizes the key pathogenic mechanisms underlining the relationship between NAFLD and reduced bone mineral density.

### 4.1. Vitamin D

The pathogenesis of the mutual influence of hepatic steatosis and bone tissue remodeling processes is a subject for discussion in many recent papers. Most authors believe that the pathogenesis of decreased BMD in NAFLD is multifactorial and, according to the available literature data, is associated with vitamin D deficiency, decreased physical activity, and the release of pro-inflammatory cytokines from the liver that affect bone [3].

The role of vitamin D deficiency in bone homeostasis is well known. It was confirmed that patients with NAFLD have lower serum levels of vitamin D, compared with otherwise healthy persons [19]. Vitamin D is known to exhibit antifibrotic activity in the liver via inhibiting the proliferation of hepatic stellate cells and the expression of profibrotic mediators, such as platelet growth factor and transforming growth factor beta (TGF-β). Similarly, vitamin D suppresses the expression of collagen, α-smooth muscle actin, and tissue inhibitors of metalloproteinase-1 (TIMP-1). Furthermore, vitamin D exhibits systemic and tissue-specific anti-inflammatory properties by reducing oxidative stress.

An animal study confirmed the antifibrotic effect of 1,25(OH)2D3 (the active form of vitamin D) on the development of NAFLD by inhibiting hepatic stellate cells, thereby suppressing the expression of TGF-β and platelet-derived growth factor [20]. An important role of vitamin D deficiency in the development of NAFLD was also shown in a study on diet-induced mice model of NAFLD. It was established that a high-fat diet in combination with vitamin D deficiency was crucial for the development of steatosis, the pathogenetic mechanisms of which in this case included systemic inflammation, changes in the gut microbiota composition, and the development of insulin resistance [21]. Additionally, vitamin D supplementation was shown to reduce the progression of NAFLD via reducing inflammation and hepatocyte apoptosis [22]. These data may be explained by the ability of an active form of vitamin D to attenuate oxidative stress, and to upregulate the expression of genes encoding antioxidant enzymes [23]. Moreover, it was shown that vitamin D supplementation led to a significant increase in B-cell lymphoma 2 (BCL2) gene expression, whereas the expression of all pro-apoptotic genes and TNF-α was decreased [24].

Increasing evidence suggests that the beneficial effect of vitamin D in NAFLD at least in part may be attributed to its impact on gut microbiota composition. Thus, in their recent study R. Thomas et al. (2020) demonstrated that the serum levels of 1,25-dihydroxyvitamin D (active form of vitamin D) were correlated with the α-diversity of gut microbiota and butyrate-producing bacteria [25]. Additionally, in a high-fat diet-induced rat model of NAFLD X-L, Zhang et al. (2023) showed that intraperitoneal injection of vitamin D promoted a decrease in hepatic lipid accumulation, along with improvement in serum transaminase activity and blood glucose profile [26]. Authors attributed this effect to the modulation of gut microbiota leading to the enhancement of tyrosine, tryptophan, and sphingolipid metabolism, as well as arginine biosynthesis.

Taking into account the specific role of vitamin D in systemic inflammation, a large number of clinical studies have focused on examining the dynamics of the liver histological picture in NAFLD following the intake of biological supplements containing the active form of vitamin D. Though recently published meta-analyses did not confirm the presence of any relationship between the intake of vitamin D and the severity of NAFLD [27,28], authors note that their data should be interpreted with caution, taking into account the low numbers of observed subjects and the lack of confounder adjustments in the included studies. They also indicate the need for further research in order to clarify this issue. Nevertheless, available evidence suggests the beneficial effects of long-term treatment with low-dose vitamin D in young patients with NAFLD without severe liver fibrosis and comorbidities [29,30,31,32,33].

### 4.2. Chronic Inflammation

Chronic inflammation, as one of the factors in NAFLD pathogenesis, seems to be involved in BMD reduction. Lipid overload associated with lipotoxicity triggers an inflammatory cascade mediated by hepatic stellate and dendritic cells, which produce a variety of proinflammatory, procoagulant, and profibrogenic molecules. The so-called *sterile inflammation*, associated with local damage, leads to liver fibrosis and inflammatory osteoporosis [34]. It is known that in patients with NAFLD, the blood concentration of TNF-α is increased; and inhibition of TNF-α, caused by the introduction of pentoxifylline, reduces inflammation of hepatocytes and normalizes liver function [35]. Tumor necrosis factor alpha also affects bone metabolism by inhibiting osteoblastogenesis and inducing osteoclasteogenesis, which causes bone loss. In addition, the presence of chronic inflammation is associated with an increase in the level of adipokines (leptin) and prooxidants (oxidized low-density lipoproteins and uric acid), as well as with a decrease in the level of anti-inflammatory cytokines (IL-10) and antioxidants (paraoxonase 1) [36]. A group of researchers led by B.L. Ilesanmi-Oyelere (2019) established higher levels of cytokines (interferon alpha-2, interferon gamma, IL-12p70, IL-33) and chemokines (monocyte chemotactic factor-1) in postmenopausal women with osteoporosis [37,38].

The chronic inflammatory process also contributes to the increased formation of reactive oxygen species. Excessive oxidative stress leads to stimulation of osteoclast differentiation, osteoblast and osteocyte apoptosis, and simultaneous suppression of osteoclast apoptosis and osteoblast differentiation, thereby affecting bone homeostasis [39,40]. These data may imply that it is chronic inflammation that links NAFLD and osteoporosis, albeit further studies are required to elucidate this relationship [35].

### 4.3. Gut Microbiota

Data accumulated over the past years suggest that there may be an inextricable relationship between bone homeostasis and gut microbiota. This issue has been investigated in a variety of pathological conditions, including but not limited to obesity [41], which is known to be strongly associated with NAFLD. It is believed that gut microbiota may impact on the bone tissue metabolism through the following mechanisms: regulation of nutrient absorption, intestinal permeability, and immune response, as well as synthesis of several metabolites (i.e., short-chain fatty acids [SCFAs]), hormones, or neurotransmitters (i.e., 5-hydroxytryptamine) [42]. Further we will briefly discuss some of these mechanisms that may be relevant for the pathogenesis of both NAFLD and osteoporosis.

*Lipopolysaccharides* (*LPS*), the cell wall component of Gram-negative bacteria, are known to be involved in the activation of the host innate immune response and were shown to play an important role in NAFLD development and progression. The latter may be due to the ability of LPS to drive and maintain systemic low-grade inflammation, also known to be involved in the disturbance of bone tissue metabolism. Lipopolysaccharides can stimulate the secretion of different pro-inflammatory cytokines, in particular IL-1β and TNF-α, thus inducing the expression of the receptor activator of nuclear factor kappa B ligand (RANKL). This results in the activation of bone resorption and inhibition of bone formation [43].

Currently, it is suggested that bile acids can be considered as important signal molecules participating in the pathogenesis of NAFLD. Gut microbiota are involved in bile acids metabolism as they promote the formation of secondary bile acids in the intestine. Moreover, through farnesoid X receptor (FXR) and G protein-coupled bile acid receptor 5 (TGR5) signaling, gut microbiota are capable of changing the amounts and type of secondary bile acids, thus influencing their metabolic effects [44]. In turn, secondary bile acids, being potent TGR-5 agonists through the stimulation of TGR5, can increase the production of glucagon-like peptide-1 (GLP-1), which can promote the secretion of calcitonin, thus inhibiting bone resorption. Additionally, this hormone can stimulate the proliferation of osteoblasts and inhibit osteoclasts [45].

Accumulated data concerning the role of SCFAs in the pathogenesis of NAFLD appear to be controversial. Some studies suggest the beneficial effect of butyrate in the attenuation of NASH due to the modulation of gut microbiota, intestinal barrier function, and the upregulation of the GLP-1 receptor expression, whereas the others indicate that acetate and propionate may promote the progression of NAFLD through the maintenance of low-degree inflammation [46]. The production of SCFAs may also be a mechanism by which gut microbiota may influence bone homeostasis. This effect is explained by the ability of SCFAs to increase synthesis and secretion of IGF-1, as well as by the anti-inflammatory action of these molecules through the effect on Tregs development and differentiation [44].

### 4.4. Diet and Physical Activity

Nowadays, lifestyle modification strategies, including the optimization of nutrition and increase in physical activity, remain the basis of NAFLD treatment. It is well-documented that 5–10% weight loss from the baseline value is associated with the resolution of liver steatosis, inflammation, and fibrosis. Moreover, physical activity itself, irrespective of nutrition modification, may have a beneficial impact on liver disease [47]. This may be explained by the hypothesis that skeletal muscle quantity and quality are of great importance for the pathogenesis of NAFLD, a possibility which is receiving more and more attention from the scientific community [48]. At the same time, taking into account that bone and muscle tissues are inextricably related to each other both anatomically and functionally, one can conclude that muscle health may also be of great importance for bone homeostasis.

A high-fat diet (HFD) is known to be implicated in NAFLD development and progression. At the same time, data presented in the literature indicate that HFD may have a negative impact on bone density, and this may be due to several mechanisms, including gut microbiota dysbiosis, immune disorders, as well as excess accumulation of adipose tissue [49]. It is established that increased dietary fat intake results in the accumulation of adipose tissue in the bone marrow [50] and promotes the differentiation of multipotent stem cells (MSCs) into adipocytes rather than osteoblasts, disturbing the bone formation [51]. Additionally, obesity is accompanied by a decrease in serum adiponectin concentration, a biologically active substance known to be involved in bone homeostasis (its effects will be discussed in more details in the next section of the review).

A high-fat diet may have a negative impact on skeletal muscle quality and function, leading to disturbed energy homeostasis and physical performance. Thus, HC Spooner et al. (2021) demonstrated that HFD was associated with increased extramyocellular fat accumulation accompanied by a decrease in intramyocellular lipid content. These changes resulted in the reduced ability of muscles to utilize fatty acids and provided further deterioration of energy homeostasis induced by HFD [52]. It is worth noting that this effect was not accompanied by any changes in body composition.

As to the influence of physical activity on bone mineral density and bone tissue metabolites, similarly to NAFLD, physical exercises may have a beneficial impact on the above-mentioned issues. One recently published systematic review indicated that physical activity appeared to be important for the prevention of osteoporosis in people aged ≥65 years. The greatest effect was demonstrated for the training programs that included multiple exercises and resistance exercises and for high-dose training programs [53]. The other systematic review demonstrated that physical activity was able to increase bone formation and decrease bone resorption biomarkers in subjects with osteoporosis [54]. However, both reviews indicated the need for further research in this area.

### 4.5. Biologically Active Substances

The list of main bone tissue metabolites and their functions, including their role in bone turnover, is presented in Table 1. Table 2 summarizes the data on the association of bone tissue metabolites with the development of liver fibrosis and metabolic disturbances. Furthermore, we will discuss each of these substances and their key effects in detail.

#### 4.5.1. Osteopontin

Osteopontin (OPN) is a negatively charged cytokine-like protein regulating extracellular matrix metabolism and playing an important role in many biological processes, such as inflammation; cell adhesion, migration, and differentiation; cell survival and apoptosis; as well as bone matrix mineralization [74,88,89,90].

The multidirectional action of this protein is associated with its ability to interact with various molecules, including cell surface receptors, such as integrin and clusters of differentiation (CD44), intracellular signaling molecules, calcium, and heparin [55,59,91]. It has been established that OPN can exist in two isoforms: intracellular (iOPN) and secreted (OPN-s). OPN is produced by bone tissue cells (osteoclasts, osteoblasts), hepatocytes, epithelial cells, pericytes, fibroblasts, lens cells, renal tubule epithelial cells, smooth muscle cells, cells of the immune system, and also by various malignant neoplasms [55,92]. Osteopontin is also found in various body fluids, including blood, urine, bile, and breast milk [88].

Studies have shown that OPN plays a role in bone metabolism and homeostasis. OPN is not only an important factor in neuron-mediated and endocrine-regulated bone mass, but is also involved in biological activities such as proliferation, migration, and adhesion of several bone-related cells, including bone marrow mesenchymal stem cells, hematopoietic stem cells, osteoclasts, and osteoblasts. OPN has been demonstrated to be closely related to the occurrence and development of many bone-related diseases, such as osteoporosis, rheumatoid arthritis, and osteosarcoma [74,89,90].

The OPN protein is encoded by the secreted phosphoprotein 1 gene (*SPP1*), which has seven exons 5000 bases long and is located on chromosome 4. Under physiological conditions, *SPP1* is abundantly expressed in the placenta, kidneys, brain, and liver. In a healthy liver, cholangiocytes exhibit the highest expression of *SPP1*, followed by macrophages, hepatocytes, sinusoidal endothelial cells, hepatic stellate cells, T cells, and natural killer cells [93].

Some studies established the role of OPN in the development of alcoholic liver disease (ALD) and NAFLD, including steatohepatitis and cirrhosis, as well as in the pathogenesis of other chronic liver diseases, such as viral hepatitis and drug-induced liver injury. In particular, it was noted that patients with NAFLD had an increased OPN level in the blood plasma, and there was a direct dependence of OPN amount on the stage of liver fibrosis [75,76].

Although the pathogenesis of cirrhosis is a multifactorial process, an essential stage of its development is the activation of liver stellate cells, which are the main source of collagen, the key component of the extracellular matrix. The process of stellate cell activation is mediated by numerous growth factors, cytokines, and chemokines. One of the signaling molecules that activate hepatic stellate cells, as well as molecules associated with cell damage and fibrosis, is OPN. It was discovered that the OPN concentration in plasma is associated with liver fibrosis—viz., it statistically significantly correlates with the stage of fibrosis as well as the presence of liver failure, portal hypertension and hepatocellular carcinoma. This allows OPN to be considered as a promising target in the treatment of chronic liver diseases and hepatocellular carcinoma [94,95,96]. 

#### 4.5.2. Procollagen Type 1 N-Terminal Propeptide

The procollagen type 1 N-terminal propeptide (P1NP) is a precursor of type 1 collagen and a marker of osteogenesis. It is known that the organic matrix of the bone is represented mainly by type 1 collagen, which is formed from type 1 procollagen synthesized by fibroblasts and osteoblasts. The P1NP is released into the intercellular space and bloodstream during the formation of type 1 collagen and its incorporation into the bone matrix. Thus, the P1NP content in the blood is directly proportional to the amount of collagen newly synthesized and incorporated into the bone tissue [60]. The production of type 1 collagen and, accordingly, P1NP increases in bone diseases accompanied by metabolic bone disorders (osteoporosis, hyperparathyroidism, Paget’s disease, metastatic lesions, and osteomalacia). In clinical practice, a blood test for P1NP is used before initiating antiresorptive therapies, as well as for assessing the treatment efficacy (it is expected that this indicator decreases in the course of therapy).

Changes in the blood concentration of P1NP are also closely associated with liver fibrosis. It was discovered that the P1NP content was statistically significantly linked with the stage of liver fibrosis in postmenopausal women with diabetes mellitus. Moreover, the probability of having severe liver fibrosis in female study subjects was 3.65 times higher even after adjusting for age, duration of diabetes, glycated hemoglobin level, smoking, waist circumference, glomerular filtration rate, the presence of dyslipidemia and arterial hypertension, and intake of metformin or thiazolidinediones. According to the results of histological examination of liver tissue samples from patients with a mean body mass index of 44 kg/m^2^, higher stages of liver fibrosis were independently associated with higher levels of P1NP in their blood [78]. Data have been published that indicate that P1NP concentration can be a marker of liver fibrosis in other chronic liver diseases as well [61,79,80], for e.g., P1NP levels were higher in patients with primary biliary cholangitis than in control subjects [79]. Similarly, patients with alcoholic cirrhosis demonstrated a significantly higher mean serum P1NP content against the control group, and its concentration seemed to increase with the severity of liver damage (i.e., P1NP level was significantly higher in patients with decompensated liver cirrhosis [Child-Pugh class C] vs. the patients with compensated liver cirrhosis [Child-Pugh class A]) [77]. The following factors could possibly account for the strong association between blood P1NP concentration and pronounced liver fibrosis in NAFLD patients: (1) P1NP metabolism occurs in the liver; (2) stellate cells are intrinsically capable of producing this propeptide. The liver of a healthy person contains mainly fibril-forming collagen types I and III. During fibrogenesis, the content of type I collagen increases eightfold. Collagen is integrated into the extracellular matrix, which leads to the development and progression of fibrosis [81]. Since both bone and fibrous liver are major sources of type I collagen metabolites, it is important to differentiate between these sources when attempting to determine the relative contributions of bone and liver to circulating P1NP concentrations [80,81].

#### 4.5.3. Osteoprotegerin

Osteoprotegerin (OPG) is a glycoprotein and one of the members of the TNF-α receptor superfamily. It plays a central role in bone turnover via inhibiting the differentiation and activation of osteoclasts and promoting their apoptosis. Osteoprotegerin prevents the receptor activator of nuclear factor kappa B (RANK) from binding to its ligand, RANKL, thereby hindering its action on cells, thus inhibiting osteoclast activation and stimulating their apoptosis. It is assumed that the state of bone tissue is largely determined by the local RANKL:OPG ratio [62,63,64,65,66,67].

Biosynthesis and secretion of OPG by osteoblasts are stimulated by various cytokines: IL-1, IL-6, and TNF-α. On the contrary, parathyroid hormone, 25-hydroxyvitamin D, and prostaglandin E2 reduce OPG expression [84]. Osteoprotegerin was initially discovered in bone tissue. Later on, information appeared concerning its detection in the vascular wall, heart, lungs, liver, kidneys, and placenta. In this regard, more and more data have recently appeared in the literature on the possible involvement of OPG in the pathogenesis of cardiovascular diseases and diseases of the liver and kidneys. For e.g., it was demonstrated that OPG is one of the key biologically active substances that determine the relationship between metabolic syndrome and cardiovascular risk [65,66,67]. Clinical studies established that OPG may also be a risk factor for progressive atherosclerotic cardiovascular disease [84,97,98,99].

A certain diagnostic value of OPG in the progression of NAFLD was established. M. Yang et al. (2016) examined the diagnostic value of determining OPG in patients with NAFLD [85]. The study involved 136 patients with NAFLD and 83 healthy volunteers. Besides general clinical examination, all study participants underwent a liver biopsy; biochemical markers of liver damage were determined, and the state of lipid metabolism was assessed. Significant correlations of the OPG level were established with the activity of alanine aminotransferase and the content of triglycerides (*p* < 0.05), as well as with morphological changes in the liver of patients with NAFLD (*p* < 0.01). The serum level of OPG was significantly lower in patients with NAFLD, compared with the control group. Further, patients with more pronounced liver fibrosis had an even lower serum content of this glycoprotein. The authors suggested that the serum concentration of OPG could be a diagnostic marker of NAFLD and could also be used to assess the rate of its progression [85]. These results were supported by another study that found lower serum OPG levels in patients with abdominal obesity, insulin resistance, and NAFLD. The serum concentration of OPG was significantly lower in patients with confirmed steatohepatitis (median: 45 pg/mL, *p* < 0.001) vs. the patients with simple steatosis (57 pg/mL, *p* < 0.001). The highest serum OPG concentrations were observed in the control group (92 pg/mL) [86].

Thus, OPG may stimulate fibrogenesis through TGFβ1 and is associated with the degree of fibrogenesis. It should therefore be investigated further as a possible drug target for liver fibrosis or as a biomarker for the treatment success of novel antifibrotics [100].

#### 4.5.4. Adiponectin

Adiponectin is an adipocytokine that is intensively expressed in human adipose tissue [68,69,70,71,72]. It is secreted almost exclusively by adipocytes, mainly in visceral adipose tissue and, to a lesser extent, in peripheral adipose tissue and bone marrow. The level of adiponectin depends on many factors, such as age, sex, body weight, and comorbidities.

Adiponectin is secreted as a monomer called “full-length” adiponectin (fAd), which is associated with forming complexes with different molecular weights circulating in the plasma. In the local microenvironment, fAd can be cleaved in the “globular form” of adiponectin (gAd) by the elastase secreted by activated monocytes, neutrophils, and macrophages, thus suggesting that the conversion of fAd into gAd could be driven by local inflammation [73].

In healthy liver, adiponectin controls the metabolism of both glucose and lipids, decreasing gluconeogenesis and stimulating glycolysis and fatty acid oxidation. These metabolic effects occur through the binding of gAd to adiponectin receptor 1 (AdipoR1) and fAd to the specific hepatic receptor AdipoR2 (adiponectin receptor 2) [73]. 

Hepatic stellate cells and Kupffer cells constitutively express the same amount of AdipoR1 and AdipoR2. Hepatic stellate cells are activated following trauma and lead to the secretion of collagen and to the formation of scar tissue, leading to chronic fibrosis or cirrhosis. Kupffer cells are key mediators of both liver injury and repair [87].

Animal-based studies have demonstrated that adiponectin possesses potent protective activities against various forms of liver injuries [101,102,103]. Although the mechanism is not yet clear, some evidence suggests that adiponectin directly opposes the damaging effects of TNFα within the liver tissue. In animal models of both alcoholic and non-alcoholic steatohepatitis, exogenous adiponectin reduces hepatomegaly, depletes lipid accumulation, quenches hepatic inflammation, and decreases hepatic expression and plasma concentrations of TNFα, thus suggesting that adiponectin may counteract hepatic lipid accumulation through the antagonism of TNFα [73].

It is known that the concentration of adiponectin is inversely associated with the presence and severity of NAFLD. A correlation was established between a decrease in the concentration of glutathione and adiponectin in individuals with NAFLD and/or T2DM. Lower levels of adiponectin are observed in metabolic syndrome, T2DM, insulin resistance, and dyslipidemia. In their study, T. Yatagai et al. (2003) demonstrated that low blood levels of adiponectin in men with T2DM were associated with the accumulation of visceral (rather than subcutaneous) fat [69], while N. Sandhya et al. (2010) revealed an association between increased oxidative stress and low adiponectin concentrations in NAFLD patients with and without T2DM [70]. 

Adiponectin also exhibits anti-osteoporotic activity, promoting differentiation and mineralization of osteoblasts and inhibiting the secretion of OPG [100]. Adiponectin stimulates the expression of osteocalcin, which acts as a hormone regulating glucose metabolism and fat mass [72,73,87,101,102,103,104,105,106]. The potential effect of adiponectin on osteoblasts and osteoclasts, and therefore on bone remodeling, may be mediated through the endocrine system and may influence lipid metabolism. Some authors noted that adiponectin could be an independent predictor of BMD, and its concentration positively correlated with biochemical markers of bone metabolism in postmenopausal women, but not in premenopausal women [107]. There seem to be associations between the concentration of sex hormones, the metabolism of adiponectin, the production of proinflammatory factors, and the transition to menopause [108,109,110,111,112]. Other reports indicated that the overall level of adiponectin in postmenopausal women was significantly lower in the presence of obesity. This finding suggests that adiponectin is a potential biomarker for osteoporosis in obese postmenopausal women. Adiponectin is also considered as a factor capable of inducing the proliferation and differentiation of osteoblasts through its interaction with the AdipoR1. As a result of adiponectin interaction with its receptor, a temporary dose-dependent increase in the activity of alkaline phosphatase, osteocalcin, and the production of type I collagen are observed. Thus, one can suggest that high levels of adiponectin increase BMD and activate osteoblast differentiation [77].

The main effects of treatment with bone tissue metabolites, their analogs, and agonists/antagonists of their receptors are described in Table 3.

## 5. Conclusions

The results of numerous clinical studies conducted in recent years confirmed the presence of a clinical and pathogenetic relationship between NAFLD and osteoporosis. However, it should be taken into account that most of the studies in this field included mainly elderly and senile patients. Hence, the features of the pathogenesis of osteopenia and osteoporosis in NAFLD still remain insufficiently studied. For instance, there is virtually no information in the published sources on the significance of bone tissue metabolites for the diagnosis of osteopenia and osteoporosis in young and middle-aged people suffering from NAFLD. Most studies on the relationship between NAFLD and bone disease were conducted in the Asia-Pacific region, and the prevalence of osteopenia and osteoporosis in the NAFLD cohort of the European region remains largely unknown. At the same time, given the medical and social significance of NAFLD, lack of effective medicamentous treatment for the disease, and frequent association of NAFLD with ailments of various organs and systems, it seems relevant to search for reliable diagnostic and prognostic markers of the disease and for new therapy targets. Additionally, this necessitates the active detection of NAFLD and stratification of patients based on the risk of disease progression and poor prognosis, which is largely determined by concomitant extrahepatic factors in NAFLD, in particular, by the risk of cardiometabolic complications. In this respect, bone tissue metabolites represent promising candidates. Considering that obese patients, in whom NAFLD prevalence reaches nearly 85%, could develop sarcopenia at any age, it can be assumed that this will be true for both osteopenia and osteoporosis, since metabolism, functional capacity, and skeletal muscle condition are closely related to bone health. Hence, further study of osteopenia, osteoporosis, and sarcopenia in NAFLD, including in young and middle-aged patients, is of great importance and certainly deserves future attention.

## Figures and Tables

**Table 1:** Main bone tissue metabolites and their functions.

Biologically Active Substance	Encoding Gene	Tissue Distribution/Main Sources	Functions	Role in Bone Tissue Metabolism	References
Osteopontin (OPN)	Secreted phosphoprotein 1 (SPP1)	Bone cells (preosteoblasts, osteoclasts/osteoblasts, osteocytes), hepatocytes, epithelial cells, endothelial cells, fibroblasts, renal tubular cells, smooth muscle cells, immune cells (T cells, macrophages, and natural killer cells), malignantly transformed epithelial cell lines	Regulation of extracellular matrix metabolism and bone biomineralization; participation in immune response, inflammation, and tissue repair	Osteopontin participates in the regulation of bone mass through the neuron- and endocrine-mediated mechanisms	[55,56,57,58,59]
N-terminal propeptide of procollagen type 1 (P1NP)	type 1 collagen (COL1)	Osteoblasts, hepatic stellate cells	N-terminal and C-terminal propeptides of procollagen type 1 represent the parts of the procollagen molecule. Procollagen type 1 is secreted into the extracellular space where the N-terminal and C-terminal propeptides are cleaved by propeptidases, generating the basic functional heterotrimeric unit of collagen 1. Collagen 1 contributes around 90% of the total organic component of bone matrix	The dynamic and sensitive marker of changes in bone formation	[60,61]
Osteoprotegerin (OPG)	TNF receptor superfamily member 11b (TNFRSF11B)	Osteoblasts, medullary thymic epithelial cells, intestinal microfold cells, fibroblasts, vascular endothelial cells, and some cancer cells (e.g., breast and prostate cancers)	Participation in bone homeostasis and regulation of calcium-phosphorus metabolism. It also plays a role in lymphnode organogenesis, immune modulation, fibrosis, angiogenesis, and vascular calcification. Currently the diagnostic, prognostic, and therapeutic value of osteoprotegerin in various cancers, especially bone metastasis, is widely discussed in the literature.	Osteoprotegerin is a negative regulator of bone resorption. It performs its function primarily through the impact on osteoclast development, activation, and apoptosis	[62,63,64,65,66,67]
Adiponectin	Adiponectin, C1Q and collagen domain containing	Exclusively adipose tissue, mainly visceral adipose tissue and to a lesser extent subcutaneous adipose tissue	Functions as a homeostatic factor for regulating glucose levels, lipid metabolism, and insulin sensitivity through its anti-inflammatory, anti-fibrotic, and antioxidant effects	Adiponectin may have a pro-osteogenic role by stimulating osteoblast differentiation and activity, alongside inhibiting the osteoclastogenesis.	[68,69,70,71,72,73]

**Table 2:** Association of bone tissue metabolites with the development of liver fibrosis and metabolic disturbances.

Biologically Active Substance	Possible Role in Liver Diseases	Association with Metabolic Syndrome	References
Osteopontin (OPN)	Participation in liver fibrosis development and progression via activation of stellate cells	Elevated serum level of OPN is associated with metabolic syndrome and its different components (systolic and diastolic blood pressure, fasting blood glucose, serum concentrations of total cholesterol, low density lipoprotein cholesterol [LDL-c], triglycerides, HbA1c, fasting plasma insulin, and homeostatic model assessment of insulin resistance [HOMA-IR])	[74,75,76,77]
N-terminal propeptide of procollagen type 1 (P1NP)	Production of type 1 collagen during liver fibrosis increases up to 8-fold. The P1NP seems to be related to liver fibrosis development and progression in NAFLD and some other chronic liver diseases, such as primary biliary cholangitis, and alcoholic liver disease.	Serum concentration of P1NP appears to be higher in patients with metabolic syndrome, compared to healthy subjects. At the same time, it was shown that elevated serum levels of P1NP were negatively correlated with triglyceride levels.	[61,78,79,80,81,82,83]
Osteoprotegerin (OPG)	High serum osteoprotegerin levels are associated with liver fibrosis. Elevated serum osteoprotegerin levels were shown in patients with alcoholic liver fibrosis, primary biliary cholangitis, and viral cirrhosis compared to respective controls. Osteoprotegerin may stimulate fibrogenesis in the liver through transforming growth factor beta 1 (TGFβ1) and is associated with the degree of fibrogenesis.	Serum levels of osteoprotegerin are increased in patients with metabolic syndrome. High serum levels of osteoprotegerin are associated with the poor control of type 2 diabetes mellites and the development of its complications. It was shown that osteoprotegerin levels were negatively correlated with body weight, waist circumference, HOMA-IR, and fasting plasma insulin, whereas they were positively correlated with insulin sensitivity. Taken together these data suggest that osteoprotegerin may serve as an independent predictor of metabolic syndrome.	[62,84,85,86]
Adiponectin	Adiponectin has been demonstrated to have an anti-fibrotic action in the liver by blocking the activation of the expression of pro-fibrotic genes. Increased circulating adiponectin levels were found to be associated with the development of liver fibrosis. In contrast, both serum levels and hepatic adiponectin receptor expression were shown to be decreased in NAFLD. Adiponectin reduces the transport of fatty acids and the accumulation of triglycerides in the liver.	Adiponectin induces an increase in circulating insulin levels, promotes the consumption of glucose by muscle cells and adipocytes, and inhibits the formation of glucose and glycogen in the liver and skeletal muscles. It also controls lipid metabolism by promoting the transport of fatty acids and β-oxidation in muscle cells by inhibiting hepatic lipogenesis and by stimulating the storage function of adipose tissue.	[69,73,87]

**Table 3:** Studies assessing the effects of treatment with bone tissue metabolites, their analogs, and agonists/antagonists of their receptors.

Biologically Active Substance	Analog of the Substance, Agonist/Antagonist of Its Receptor Used for the Treatment	Animal Species/Study Population	Main Findings	Reference
Adiponectin	AdipoRon (an orally active synthetic adiponectin receptor agonist)	Wild-type mice, Adipor1^−/−^, Adipor2^−/−^, Adipor1^−/−^ Adipor2^−/−^ knock-out mice, and the db/db mice	AdipoRon ameliorated insulin resistance and glucose intolerance in mice fed a high-fat diet. AdipoRon ameliorated diabetes of genetically obese rodent model db/dbmice and prolonged the shortened lifespan of db/db mice on a high-fat diet.	[102]
		27 patients with T2DM and 6 healthy controls C57BLKS/J *db*/*db* mice	AdipoR1/AdipoR2 expression was significantly decreased in the glomerulus of human diabetic kidneys compared with that of nondiabetic control kidneys, even in the earliest chronic kidney disease stage. AdipoRon ameliorated diabetes-induced renal damage by reducing intrarenal lipotoxicity and oxidative stress	[103]
		Huoyan geese	AdipoRon could alter the expression of lipid metabolism-related genes, inflammatory factors, apoptosis and autophagy genes, and adiponectin and its receptor genes in liver tissues; reduce the lipid content in blood and liver tissues of geese fed high-fat diets; improve liver antioxidant capacity; regulate apoptosis and autophagy of hepatocytes; and reduce liver inflammatory injury	[113]
	JT003 (AdipoR1/AdipoR2 dual agonist)	C57BL/6J mice	Adiponectin-based agonist JT003 potently improves insulin resistance in high fat diet induced NASH mice. JT003 treatment significantly improves endoplasmic reticulum–mitochondria axis function, which contributes to the reduced hepatic stellate cell activation in CCl4-induced liver fibrosis	[101]
	ADP355 (adiponectin analog, capable of mimicking its active site)	C57BL/6J mice	ADP355 is a potent anti-fibrotic agent that attenuates CCl4-induced liver fibrosis in mice	[114]
Osteopontin (OPN)	Mesalazine (osteopontin inhibitor)	Wistar rats	In rats with TAA-induced liver fibrosis, mesalazine exerted an anti-fibrotic action through limiting the oxidative damage and altering the TNF-ɑ pathway, along with downregulating transforming growth factor β1 (TGF-β1), OPN, alpha-smooth muscle actin, and caspase-3 signaling pathways in the liver	[115]
		Wild-type and OPN-knockout (Opn(−/−)) mice	Osteopontin delays liver fibrosis resolution following TAA cessation in the livers of wild-type mice due to sustained fibrillar collagen-I deposition. Inhibiting OPN could be an effective therapeutic strategy for resolving liver fibrosis.	[116]
Osteoprotegerin (OPG)		Balb/c mice C57BL/6 mice Cirrhotic human liver tissue	Liver fibrosis is accompanied by a higher production of OPG in liver tissue, particularly in response to TGFβ1. Hepatic stellate cells and scar-associated myofibroblasts appear to be an important source of OPG in human tissue, while in murine liver tissue (mouse model of CCl4-induced liver fibrosis) scar-associated cells appear to be the main source. Osteoprotegerin has profibrotic abilities through neutralization of RANKL and/or TRAIL and upregulation of TGFβ1 expression. Spontaneous or drug-induced resolution of fibrosis is accompanied by lower expression of OPG.	[100]

## Data Availability

Not applicable.
